# Supplementary material for: miR‐181a/b downregulation exerts a protective action on mitochondrial disease models
Source: EMBO Mol Med. 2019 Apr 12;11(5):e8734. doi: 10.15252/emmm.201708734 (PMC6505685; doi:10.15252/emmm.201708734)
Supplement: Supplementary file 1 — Appendix [file EMMM-11-e8734-s001.pdf]

Appendix

miR-181a/b downregulation exerts a protective action on Mitochondrial Disease models

Alessia Indrieri, Sabrina Carrella, Alessia Romano, Alessandra Spaziano, Elena Marrocco, Erika Fernandez-Vizarra, Sara Barbato, Mariateresa Pizzo, Yulia Ezhova, Francesca M. Golia, Ludovica Ciampi, Roberta Tammaro, Jorge Henao-Mejia, Adam Williams, Richard A. Flavell, Elvira De Leonibus, Massimo Zeviani, Enrico M. Surace, Sandro Banfi, Brunella Franco.

Table of Contents

Appendix Figure S1. The *miR-181a/b-1* cluster accounts for most of miR-181a and miR-181b expression in mouse eye.

Appendix Table S1: *p*-values Figure 5G calculated by Two-ANOVA Repetaed Measures with post-hoc analysis
Appendix Table S2: Primer sequences for q-RT-PCR analysis
Appendix Table S3: Primer sequences for human 3'UTR amplification and mutagenesis

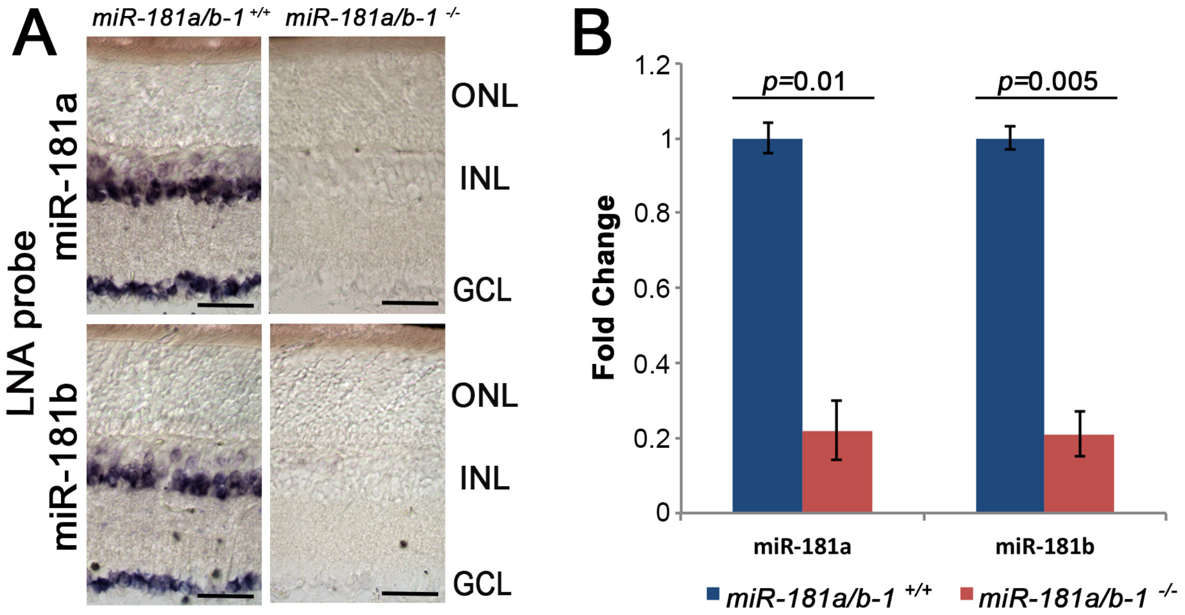

Appendix Figure S1. The *miR-181a/b-1* cluster accounts for most of miR-181a and miR-181b expression in mouse eye. A) RNA in-situ hybridization with miR-181a and miR-181b probes in retina sections of *miR-181a/b-1*$^{+/+}$ and *miR-181a/b-1*$^{-/-}$ mice. Scale bars are 50µm. B) Expression analysis of miR-181a and miR-181b in *miR-181a/b-1*$^{+/+}$ and *miR-181a/b-1*$^{-/-}$ eyes by Taqman assays. Targeted deletion of the miR-181a/b cluster-1 leads to a striking reduction of the expression levels of the mature forms of miR-181a and miR-181b. N=3; *p*-values were calculated by one tailed Student's t-test; Error bars are SEM.

Appendix Table S1: ***p*-values Figure 5G calculated by Two-ANOVA Repetaed Measures with post-hoc analysis**

**Genotype**	**log cd.s/m2**	***p*-value**
Ndufs4$^{-/-}$/*miR-181a/b-1*$^{-/-}$ *vs*. *miR-181a/b-1*$^{-/-}$	0.0001	0.99
Ndufs4$^{-/-}$/*miR-181a/b-1*$^{-/-}$ *vs*. *Ndufs4*$^{-/-}$	0.0001	0.99
Ndufs4$^{-/-}$/*miR-181a/b-1*$^{-/-}$ *vs*. WT	0.0001	0.99
miR-181a/b-1$^{-/-}$ *vs*. *Ndufs4*$^{-/-}$	0.0001	0.99
miR-181a/b-1$^{-/-}$ *vs*. WT	0.0001	0.99
Ndufs4$^{-/-}$ *vs*. WT	0.0001	0.99
Ndufs4$^{-/-}$/*miR-181a/b-1*$^{-/-}$ *vs*. *miR-181a/b-1*$^{-/-}$	0.1	0.003
Ndufs4$^{-/-}$/*miR-181a/b-1*$^{-/-}$ *vs*. *Ndufs4*$^{-/-}$	0.1	0.91
Ndufs4$^{-/-}$/*miR-181a/b-1*$^{-/-}$ *vs*. WT	0.1	0.000003
miR-181a/b-1$^{-/-}$ *vs*. *Ndufs4*$^{-/-}$	0.1	0.003
miR-181a/b-1$^{-/-}$ *vs*. WT	0.1	0.16
Ndufs4$^{-/-}$ *vs*. WT	0.1	0.000006
Ndufs4$^{-/-}$/*miR-181a/b-1*$^{-/-}$ *vs*. *miR-181a/b-1*$^{-/-}$	1	0.01
Ndufs4$^{-/-}$/*miR-181a/b-1*$^{-/-}$ *vs*. *Ndufs4*$^{-/-}$	1	0.00004
Ndufs4$^{-/-}$/*miR-181a/b-1*$^{-/-}$ *vs*. WT	1	0.000002
miR-181a/b-1$^{-/-}$ *vs*. *Ndufs4*$^{-/-}$	1	<0.0E-7
miR-181a/b-1$^{-/-}$ *vs*. WT	1	0.07
Ndufs4$^{-/-}$ *vs*. WT	1	<0.0E-7
Ndufs4$^{-/-}$/*miR-181a/b-1*$^{-/-}$ *vs*. *miR-181a/b-1*$^{-/-}$	10	0.000002
Ndufs4$^{-/-}$/*miR-181a/b-1*$^{-/-}$ *vs*. *Ndufs4*$^{-/-}$	10	0.0002
Ndufs4$^{-/-}$/*miR-181a/b-1*$^{-/-}$ *vs*. WT	10	0.000002
miR-181a/b-1$^{-/-}$ *vs*. *Ndufs4*$^{-/-}$	10	<0.0E-7
miR-181a/b-1$^{-/-}$ *vs*. WT	10	0.93
Ndufs4$^{-/-}$ *vs*. WT	10	<0.0E-7
Ndufs4$^{-/-}$/*miR-181a/b-1*$^{-/-}$ *vs*. *miR-181a/b-1*$^{-/-}$	20	<0.0E-7
Ndufs4$^{-/-}$/*miR-181a/b-1*$^{-/-}$ *vs*. *Ndufs4*$^{-/-}$	20	0.0008
Ndufs4$^{-/-}$/*miR-181a/b-1*$^{-/-}$ *vs*. WT	20	0.0009
miR-181a/b-1$^{-/-}$ *vs*. *Ndufs4*$^{-/-}$	20	<0.0E-7
miR-181a/b-1$^{-/-}$ *vs*. WT	20	0.94
Ndufs4$^{-/-}$ *vs*. WT	20	<0.0E-7
Ndufs4$^{-/-}$/*miR-181a/b-1*$^{-/-}$ *vs*. *miR-181a/b-1*$^{-/-}$	PHOTOPIC	0.39
Ndufs4$^{-/-}$/*miR-181a/b-1*$^{-/-}$ *vs*. *Ndufs4*$^{-/-}$	PHOTOPIC	0.04
Ndufs4$^{-/-}$/*miR-181a/b-1*$^{-/-}$ *vs*. WT	PHOTOPIC	0.31
miR-181a/b-1$^{-/-}$ *vs*. *Ndufs4*$^{-/-}$	PHOTOPIC	0.004
miR-181a/b-1$^{-/-}$ *vs*. WT	PHOTOPIC	0.98
Ndufs4$^{-/-}$ *vs*. WT	PHOTOPIC	0.04

Appendix Table S2: Primer sequences for q-RT-PCR analysis.

**Transcript**	**Forward**	**Reverse**
hsa-*HPRT*	AACACCCTTTCCAAATCCTCA	TGGCGTCGTGATTAGTGATG
hsa-*GAPDH*	ATGTTCGTCATGGGTGTGAA	AGGGGTGCTAAGCAGTTGGT
hsa-*PPARGC1A*	GCTTGGCCCTCTCAGACTCT	GCCACTACAGACACCGCACG
hsa-*NRF1*	GAGTGATGTCCGCACAGAAG	GTTTGCGTTTGCTGATCTTC
hsa-*COX11*	GCTGCCGTACCCCTTTATCG	CACTGGAGACTTGCATGCAC
hsa-*COQ10*	GGCGTTTTAGCCCAGGTCTTC	GCTACCATCTGCTTCACAAC
hsa-*PRDX3*	CCCGAGACTACGGTGTGCTG	CACTGGGAGATCGTTGACGC
mmu-*Hprt*	AGCTTGCTGGTGAAAAGGAC	GTCAAGGGCATATCCACAAC
mmu-*Gapdh*	GGTGCTGAGTATGTCGTGGA	CTAAGCAGTTGGTGGTGCAG
mmu-*Bcl2*	ACAACATCGCCCTGTGGATG	GTTTGTCGACCTCACTTGTG
mmu-*Mcl1*	GCTTCATCGAACCATTAGCAG	CCAGCAGCACATTTCTGATG
mmu-*Xiap*	GGTCCTGATTGCAGATCTTG	GTCAACTGCTTCTGCACAC
mmu-*Atg5*	GCCTATATGTACTGCTTCATC	CAACGTCAAATAGCTGACTC
mmu-*Erk2*	CACCAACCTCTCGTACATCG	GTGCCCGGATGATGTCATTG
mmu-*Park2*	CCATCAAGAAGACCACCAAG	CAAGTGACATCTCTCTCTAC
mmu-*Nrf1*	CTTACTGGAGTCCAAGATGC	GGAGCCAACAGAATCCTTTC
mmu-*Cox11*	GAATCCTACTGACAAACCAG	GAGGTCGACATTCACCATTC
mmu-*Coq10*	GATGATCATGGCAGCTCGGA	CTCGCACAGATCTCTTTAGG
mmu-*Prdx3*	GGAGTATTTCTGCCTCAACAG	CTCTCCATTGACAACAGCAG
mmu-*Ppargc1a*	GGAATGCACCGTAAAATCTGC	TTCTCAAGAGCAGCGAAAGC
mmu-*Sqstm1*	TTCTTTTCCCTCCGTGCTC	GGATCCGAGTGTGAATTTCC
mmu-*Map1lc3b*	TTATAGAGCGATACAAGGGGGAG	CGCCGTCTGATTATCTTGATGAG
ol-*hprt*	CTGAACAGGAACAGCGACC	TGAGGAGCTCCAATAACGTC
ol-*gapdh*	CGGCAAGCTGATAGTCGATG	AGAAACACTCCGGTGGACTC
ol-*bcl2*	GGATGACGGAGTATTTAAACG	GGCCGAAGACAGTCTTGATG
ol-*mcl1*	CGCGAAAAGAGTGGTGAAC	CCTTCAAGGACTGACACAC
ol-*atg5*	CAGATGACAAAGACGTGCTG	CTCCACATCTTCTGTCCTCA
ol-*erk2*	GCAGCGACAGCAGATAGTTC	GCCGAGATGTTGTCCAACAG
ol-*cox11*	CACCGACAAACCCATCATC	GAATTCCGGGTCGATGTAG
ol-*coq10*	GTTCTGCAAGGCCAAACTG	CGTCTCCAAGTGGTTGAAG
ol-*prdx3*	GTCCTGTTCTTCTACCCTC	AGTGAGAATCCACAGACACC
ol-*ppargc1a*	GGACGGATTGCCTTCATTTG	GTTTGCAGGTGCCAGAAGG

Appendix Table S3: Primer sequences for human 3'UTR amplification and mutagenesis

**Transcript**	**Primer sequences**
hsa-*NRF1-3'UTR*	Fw-GCTCTAGAGACTTCTTTCTGCGGAAATG<br>Rv-GCTCTAGACTGTTTTCTATGGCCAGGTG
hsa-*NRF1-3'UTR* mutagenesis	Fw-CCACAGGCAGATGCGCGTCTTGAAAGCTCCCGGGCC<br>Rv-GGCCCGGGAGCTTTCAAGACGCGCATCTGCCTGTGG
hsa-*PPARGC1A-3'UTR*	Fw-GCTCTAGAGCACTACAGATATCATATTGAGG<br>Rv-GCTCTAGATAGATTTGAAACATTCGTTTCCC
hsa-*PPARGC1A-3'UTR* mutagenesis	Fw-CTGAGCTAATAAAGGGAAACGCCGGTTTCAAATCTCTAGGTCGGG<br>Rv-CCCGACCTAGAGATTTGAAACCGGCGTTTCCCTTTATTAGCTCAG
hsa-*COX1-3'UTR*	Fw-GCTCTAGAGCCTAGCTAGAATATATGAC<br>Rv-GCTCTAGACACAGGCTTCCTACATTTAG
hsa-*COX11-3'UTR* mutagenesis	Fw-CAAGTCCATGCGCGTTAAAATGTACAGGTGGGATTG<br>Rv-CAATCCCACCTGTACATTTTAACGCGCATGGACTTG
hsa-*COQ10-3'UTR*	Fw-GCTCTAGAGCCACCTGCTTCTGACTTTAG<br>Rv-GCTCTAGACAACTTCAGTCCTTACATTG
hsa-*COQ10-3'UTR* mutagenesis	Fw-GAAGATAAGTTGGTTGGGCGTCTCCAGCACTATGCATCCC<br>Rv-GGGATGCATAGTGCTGGAGACGCCCAACCAACTTATCTTC
hsa-*PRDX3-3'UTR*	Fw-GCTCTAGACTGAGAGAAGAACCACAGTTG<br>Rv-GCTCTAGACCCTGGATTTGATAAATATCC
hsa-*PRDX3-3'UTR* mutagenesis site I	Fw-CGGTCCTGAAATTTTCATCTTGCCGGTCTTTGTATTAAACTGAATTTTC<br>Rv-GAAAATTCAGTTTAATACAAAGACCGGCAAGATGAAAATTTCAGGACCG
hsa-*PRDX3-3'UTR* mutagenesis site II	Fw-GCCGTGTAACTCCTGCAATGCCGGTTTATGTGATTGAAGC<br>Rv-GCTTCAATCACATAAACCGGCATTGCAGGAGTTACACGGC
