# Supplementary material for: miR‐181a/b downregulation exerts a protective action on mitochondrial disease models
Source: EMBO Mol Med. 2019 Apr 12;11(5):e8734. doi: 10.15252/emmm.201708734 (PMC6505685; doi:10.15252/emmm.201708734)
Supplement: Supplementary file 2 — Expanded View Figures PDF [file EMMM-11-e8734-s002.pdf]

Expanded View Figures

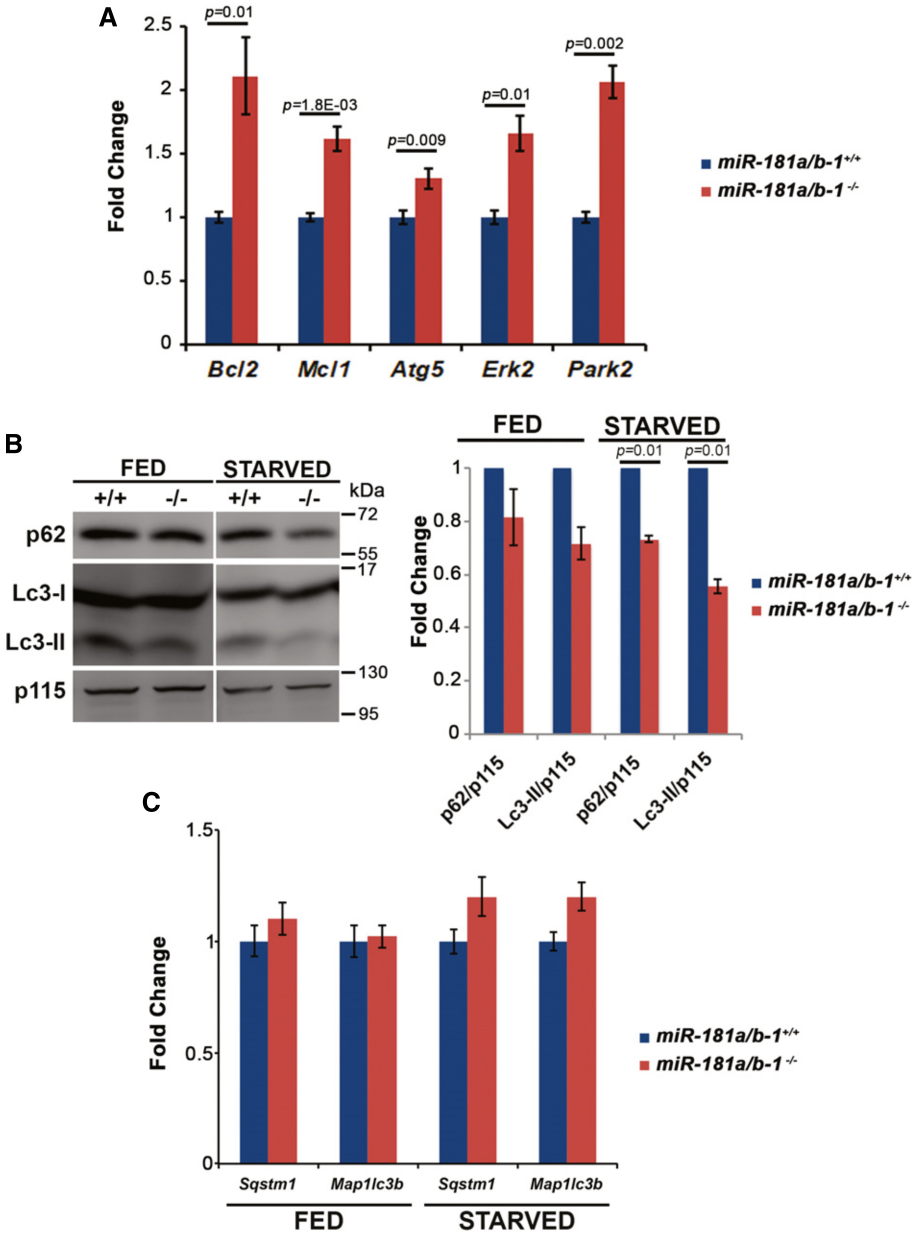

Figure EV1. ***miR-181a/b-1* deletion leads to increased autophagic flux.**

A qPCR analysis reveals increased levels of miR-181a/b targets *Bcl2*, *Mcl1*, *Atg5*, *Erk2*, and *Park2* in the eyes of *miR-181a/b-1*$^{-/-}$ versus *miR-181a/b-1*$^{+/+}$ animals. $N \geq 5$ animals/genotype.

B WB analysis (left panel) of Lc3-I/Lc3-II and p62 on protein extracts from eyes of animal in fed and starved conditions reveals decreased levels (quantified in the right panel) of both proteins in *miR-181a/b-1*$^{-/-}$ (−/−) versus *miR-181a/b-1*$^{+/+}$ (+/+) mice. $N = 2$ mice for each genotype and condition.

C qPCR analysis reveals no changes in the *Sqstm1* (p62) and *Map1lc3b* (LC3) transcript levels between *miR-181a/b-1*$^{-/-}$ and *miR-181a/b-1*$^{+/+}$ mouse eyes in both fed and starved conditions. These data indicate that the decreased levels of the autophagy markers Lc3-II and p62 observed by WB analysis are due to increased autophagic flux in *miR-181a/b-1*$^{-/-}$ eyes. $N = 3$ for each genotype and condition.

Data information: *P*-values were calculated by one-tailed Student's *t*-test; error bars are SEM.
Source data are available online for this figure.

© 2019 The Authors

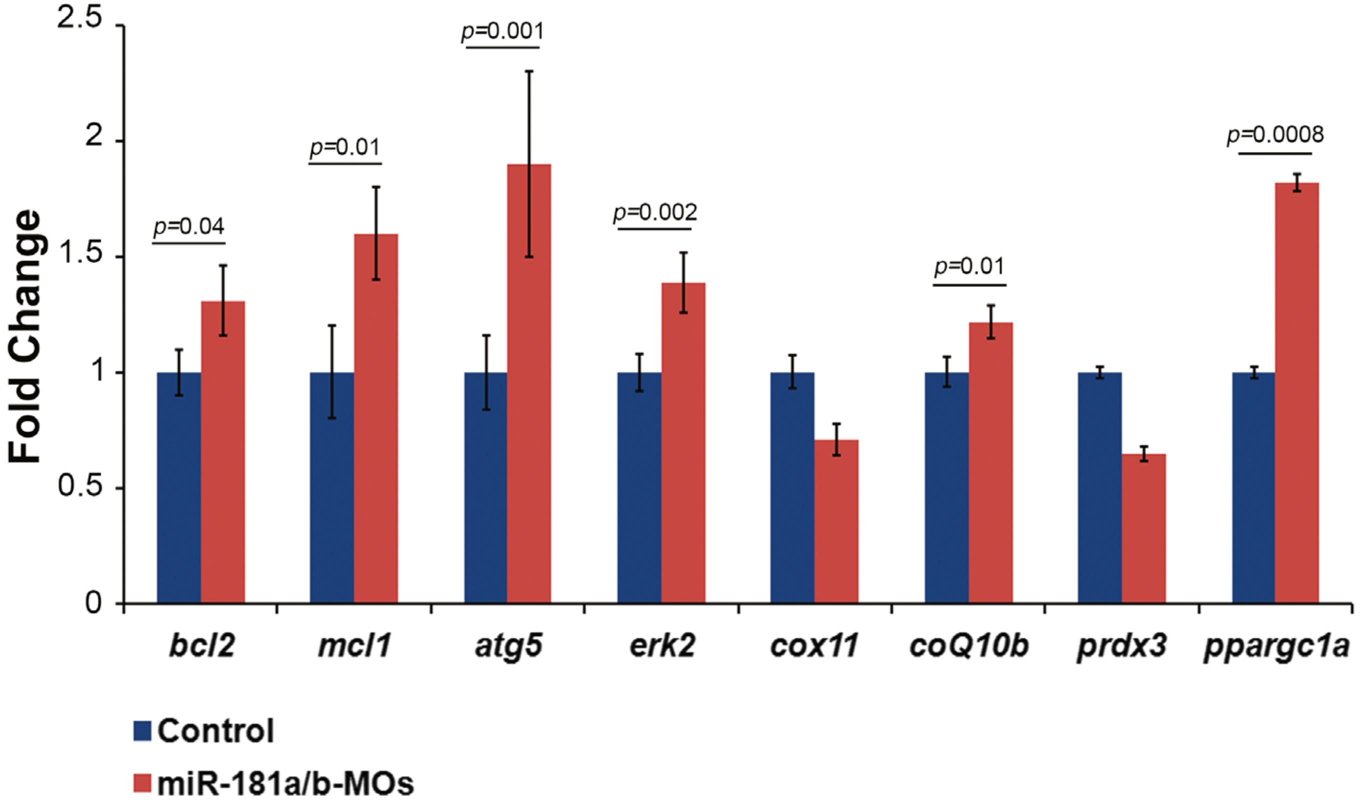

Figure EV2. miR-181a/b knockdown in medaka leads to upregulation of genes involved in mitochondrial function and autophagy.

qPCR carried out on total RNA extracted from whole miR-181a/b-MOs medaka embryos to analyze the transcript levels of the miR-181a/b targets involved in mitochondrial-dependent cell death (*bcl2*, *mcl1*), autophagy (*atg5, erk2*), and mitochondrial biogenesis and function (*cox11*, *coq10b*, *prdx3)* and of the indirect target *ppargc1a*. $N = 3$. *P*-values were calculated by one-tailed Student's *t*-test; error bars are SEM.

© 2019 The Authors

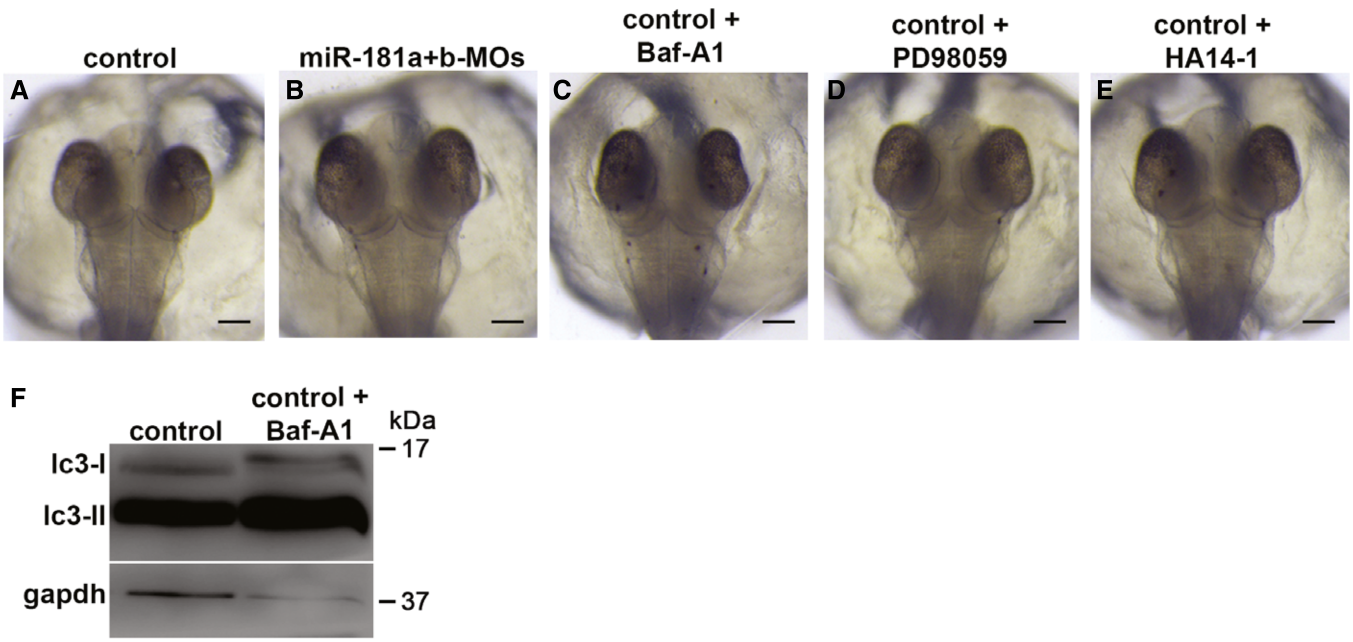

Figure EV3. Absence of abnormal phenotypes following MO-mediated silencing of miR-181a/b and drug administration.

A, B Embryos injected with miR-181a/b-MOs only (B) show no phenotypic differences compared to control embryos (A). Scale bars are 100 μm.

C–E Bafilomycin A (Baf-A1) (C), PD98059 (D), and HA14-1 (E) do not induce any morphological alterations in control embryos at the concentrations used in this study. Scale bars are 100 μm.

F The concentration of Bafilomycin A used for the study blocks autophagy as demonstrated by lc3-I/II Wb analysis.

Source data are available online for this figure.

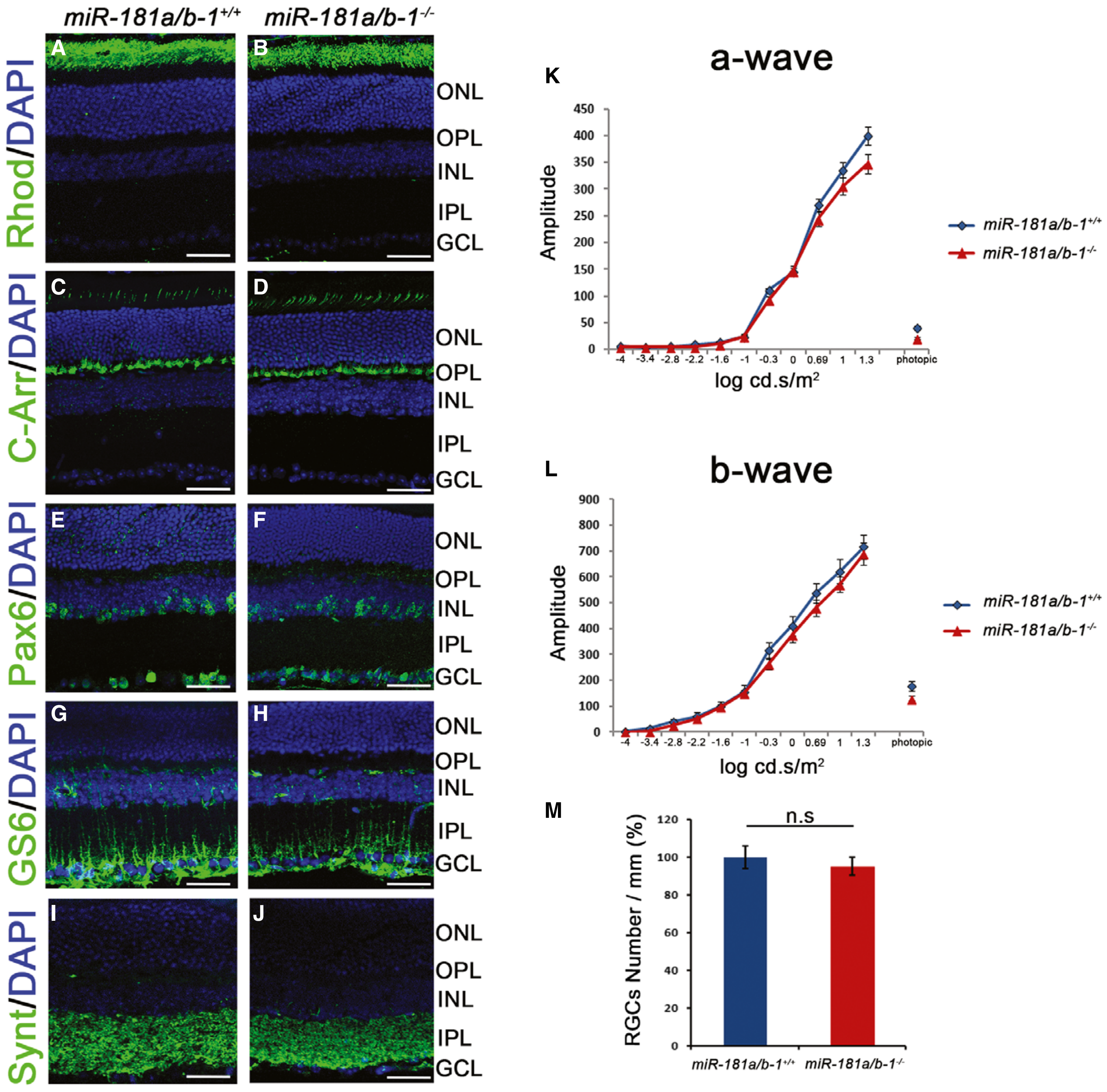

Figure EV4. *miR-181a/b-1* deletion does not cause significant alterations of retina morphology and function in mouse.

A–J Immunofluorescence staining of different retinal markers [(A, B) Rhodopsin (Rhod) for rods; (C, D) Cone arrestin (C-Arr) for cones; (E, F) Pax6 for RGCs and amacrine cells; (G, H) GS6 for Muller cells; (I, J) Syntaxin (Synt) for synaptic structures in the inner plexiform layer] reveals no abnormalities in cell layer organization in the retina of *miR-181a/b-1*$^{-/-}$ with respect to control mice. Scale bars are 50 µm.

K, L Electroretinographic analysis reveals no difference in a-wave (K) and b-wave (L) patterns between *miR-181a/b-1*$^{+/+}$ and *miR-181a/b-1*$^{-/-}$ mice, indicating that miR-181a/b ablation does not cause retinal dysfunction. *N* = 10 for each genotype.

M Graphical representation of the number of RGCs/mm (indicated as %) shows no significant differences between *miR-181a/b-1*$^{+/+}$ and *miR-181a/b-1*$^{-/-}$ mice. *N* = 10 for each genotype.

Data information: *P*-value was calculated by two-tailed Student's *t*-test; error bars are SEM. ONL, outer nuclear layer; OPL, outer plexiform layer; INL, inner nuclear layer; IPL, inner plexiform layer; GCL, ganglion cell layer.

© 2019 The Authors

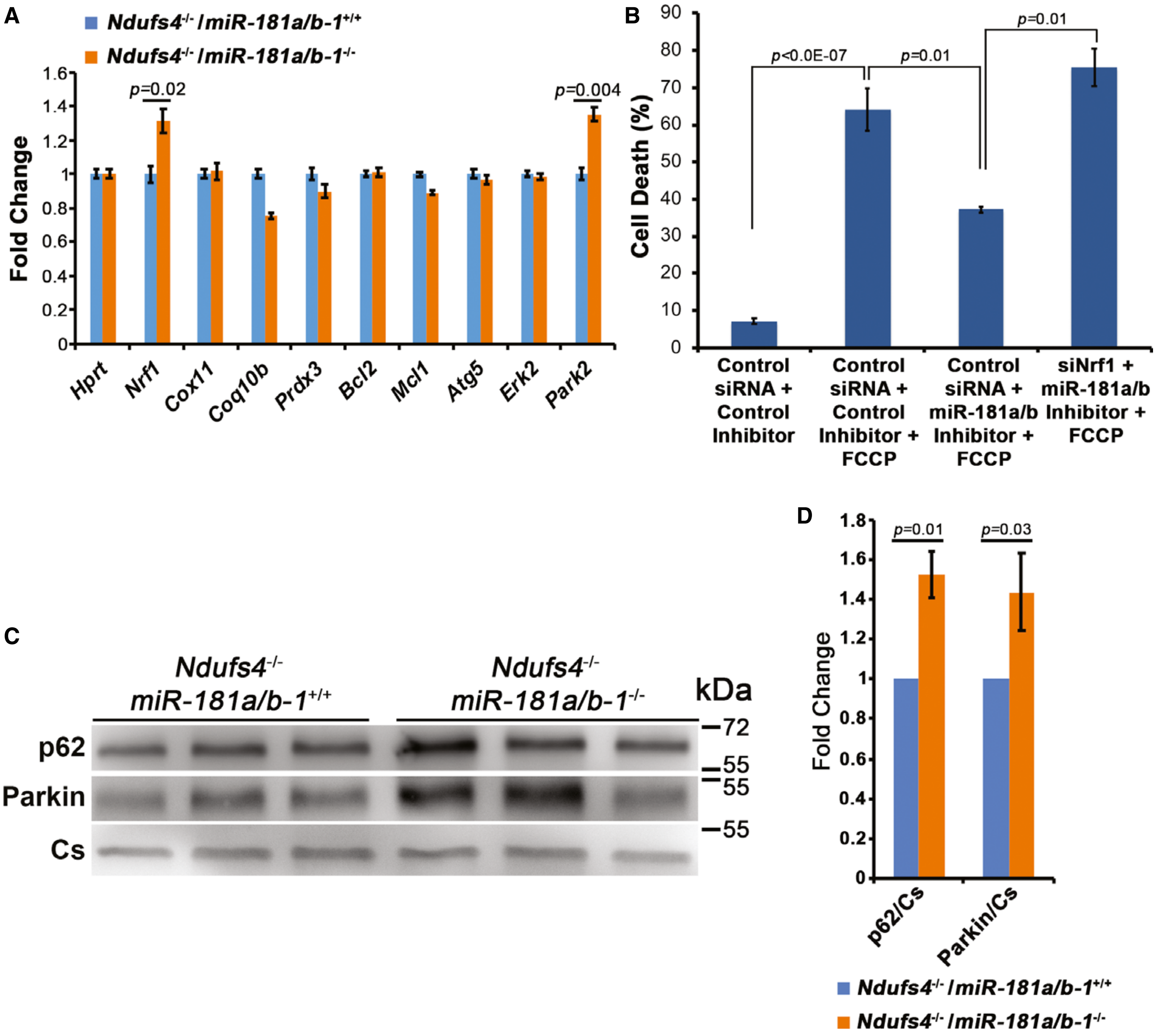

Figure EV5. miR-181a/b depletion increases *Nrf1* and *Park2* transcript levels and enhances mitophagy in *Ndufs4*$^{-/-}$ retina.

A qPCR reveals upregulation of the miR-181a/b targets *Nrf1* and *Park2* in the eyes of *Ndufs4*$^{-/-}$/*miR-181a/b-1*$^{-/-}$ versus *Ndufs4*$^{-/-}$ animals. *N* = 4 animals/genotype.

B Cell death analysis shows that *NRF1* downregulation abolishes the miR-181a/b-mediated protection in SH-SY5Y cells treated with FCCP. *N* = 4.

C, D WB analysis on mitochondrial fractions shows increased levels of p62 and Parkin (quantified in D) in mitochondrial fraction from the eye of *Ndufs4*$^{-/-}$/*miR-181a/b-1*$^{-/-}$ versus *Ndufs4*$^{-/-}$ mice. Data are normalized to citrate synthase (Cs). *N* = 3 animals/genotype.

Data information: *P*-values were calculated by one-tailed Student's *t*-test in (A), one-way ANOVA with *post hoc* analysis in (B) and one-tailed Student's *t*-test in (D). Error bars are SEM.
Source data are available online for this figure.
