# Supplementary figures and images for: miR‐181a/b downregulation exerts a protective action on mitochondrial disease models
Source: EMBO Mol Med. 2019 Apr 12;11(5):e8734. doi: 10.15252/emmm.201708734 (PMC6505685; doi:10.15252/emmm.201708734)

**FIGURE EV1B**

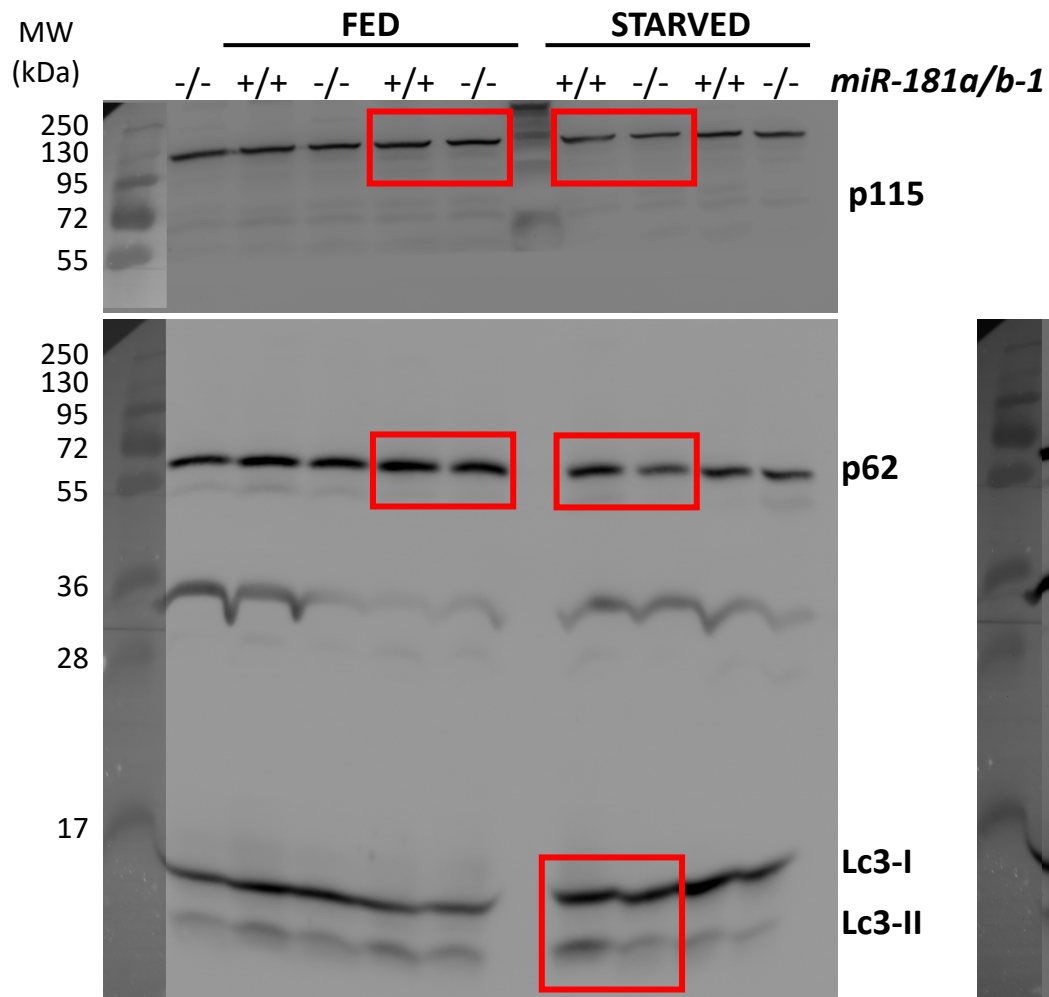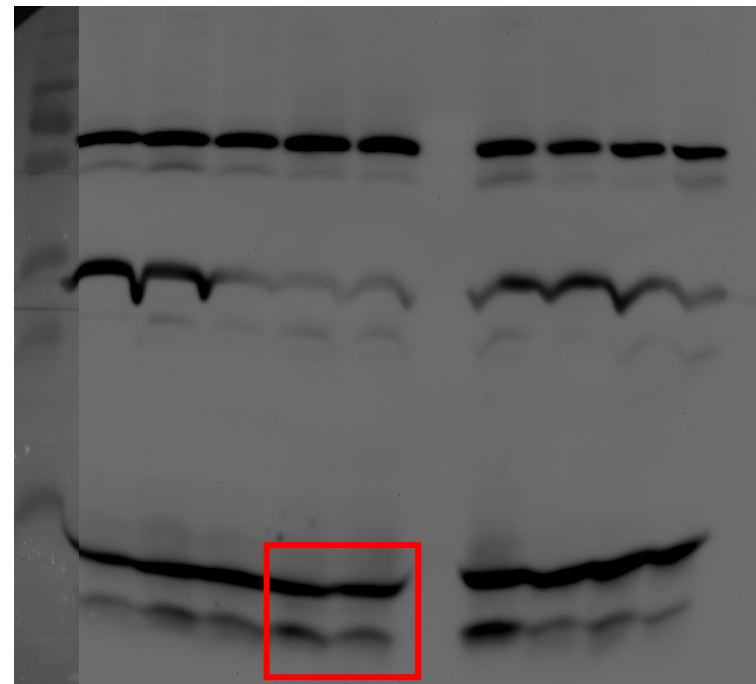

Higher exposure

Supplement: Supplementary file 3 — Source Data for Expanded View [file EMMM-11-e8734-s004.zip › 8734_EV_source_data/Source_Data_FigureEV1B.pdf]

**FIGURE EV3F**

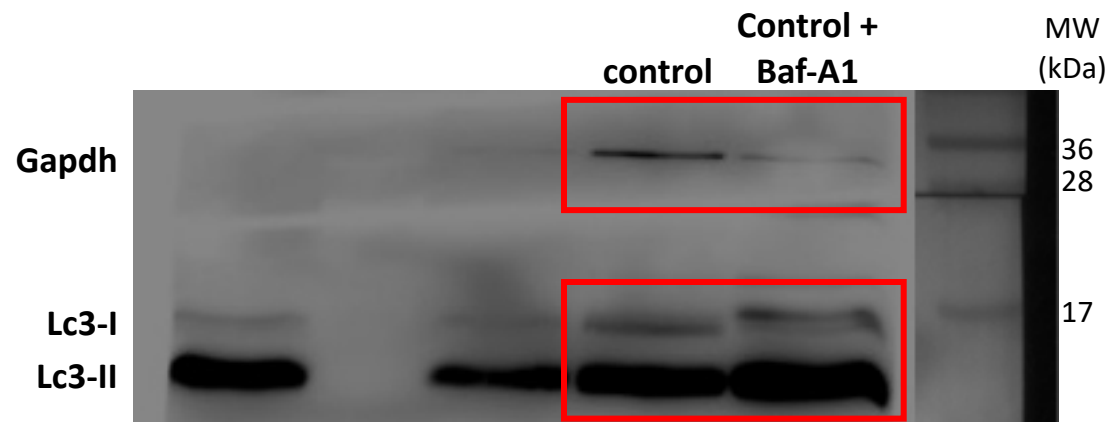

Supplement: Supplementary file 3 — Source Data for Expanded View [file EMMM-11-e8734-s004.zip › 8734_EV_source_data/Source_Data_FigureEV3F.pdf]

**FIGURE EV5C**

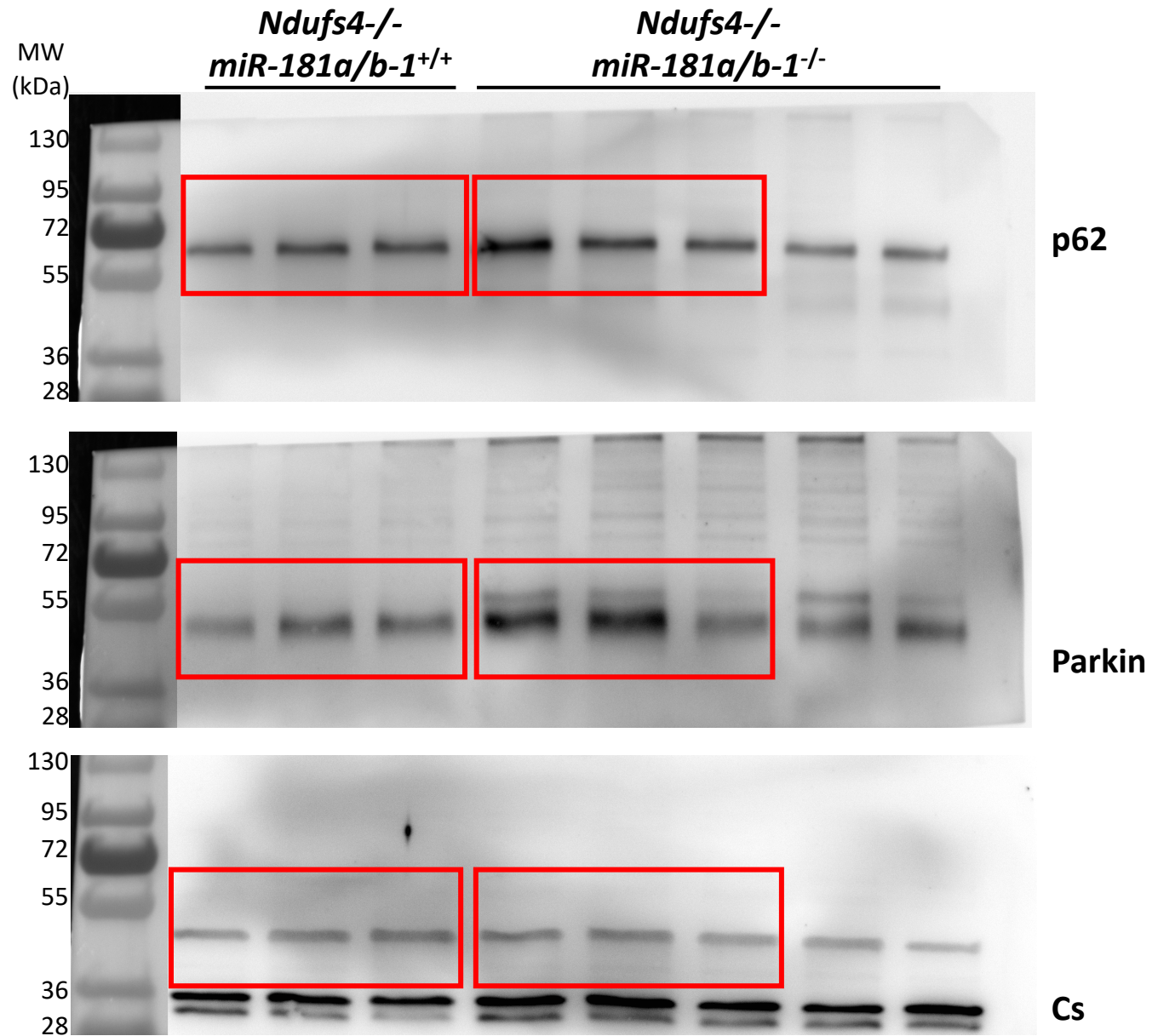

Supplement: Supplementary file 3 — Source Data for Expanded View [file EMMM-11-e8734-s004.zip › 8734_EV_source_data/Source_Data_FigureEV5C.pdf]

**FIGURE 1E**

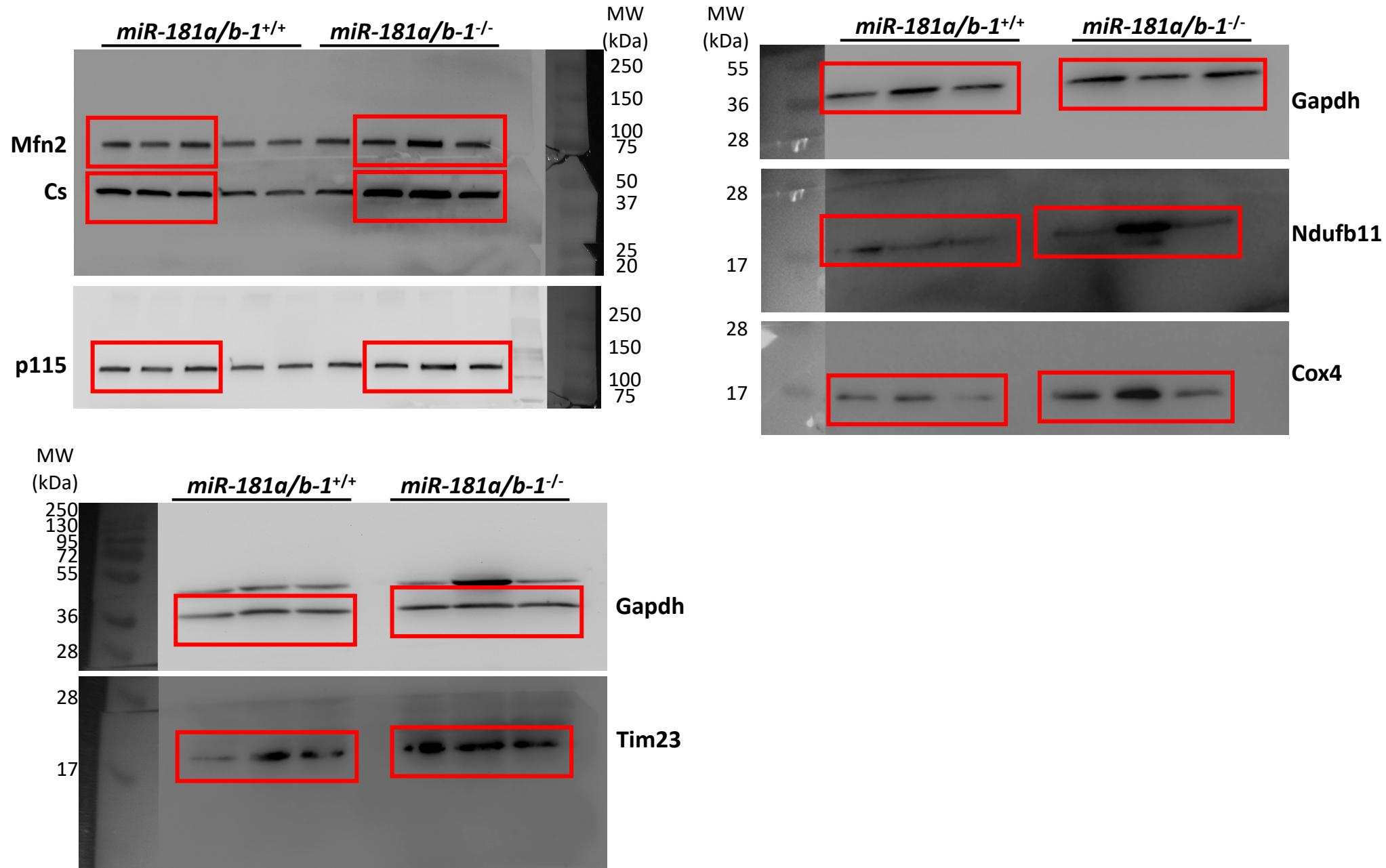

**FIGURE 1F**

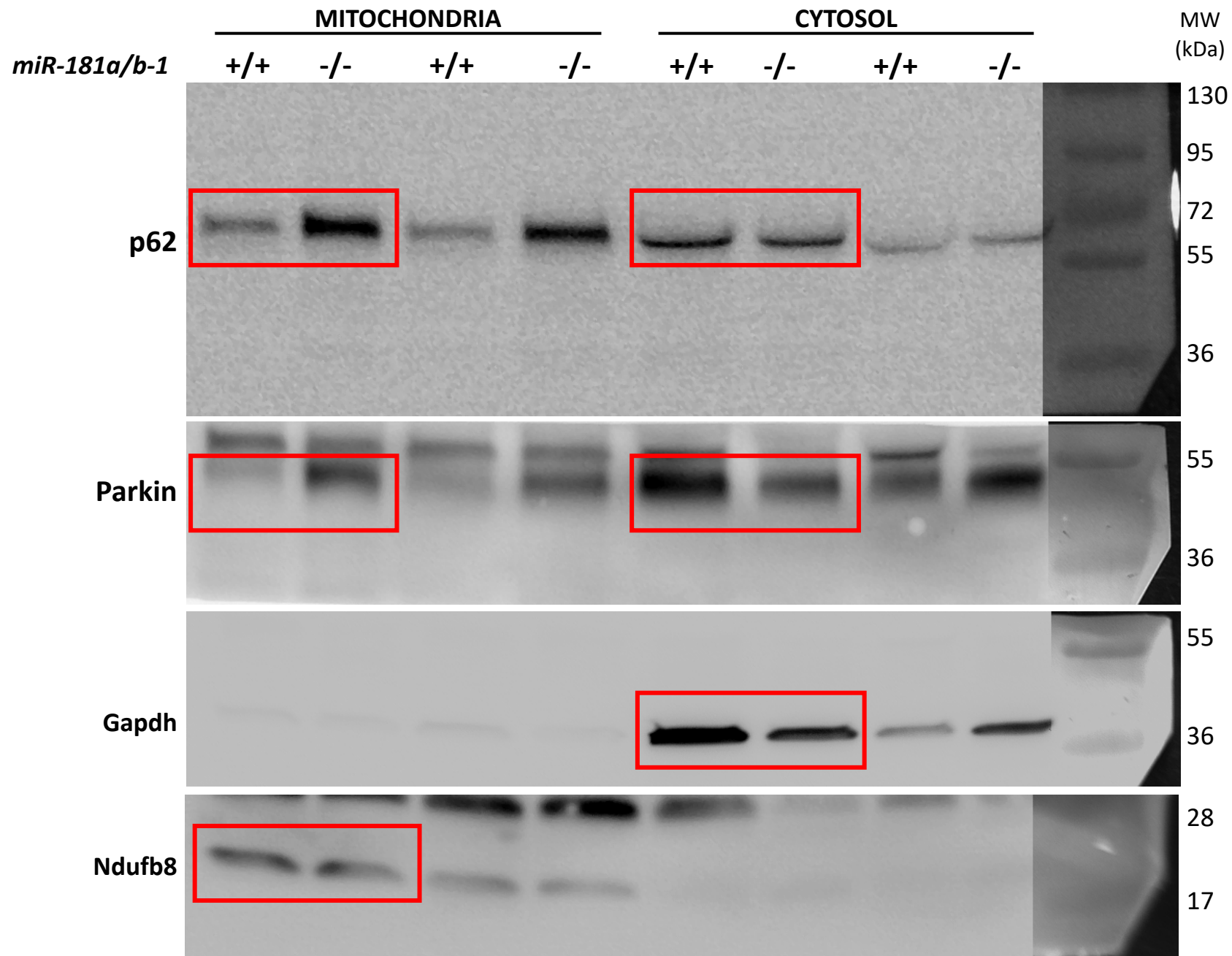

Supplement: Supplementary file 5 — Source Data for Figure 1 [file EMMM-11-e8734-s003.pdf]
